# Three-beam convergent-beam electron diffraction for measuring crystallographic phases

**DOI:** 10.1107/S2052252518012216

**Published:** 2018-10-08

**Authors:** Yueming Guo, Philip N. H. Nakashima, Joanne Etheridge

**Affiliations:** a Department of Materials Science and Engineering, Monash University, Victoria 3800, Australia; b Monash Centre for Electron Microscopy, Monash University, Victoria 3800, Australia

**Keywords:** crystallographic phase problem, three-phase invariants, convergent-beam electron diffraction, structure determination, enantiomorph ambiguity, nanocrystals, dynamical studies, multiple scattering

## Abstract

Under almost all circumstances, electron diffraction patterns contain information about the phases of structure factors, a consequence of the short wavelength of an electron and its strong Coulombic interaction with matter. However, extracting this information remains a challenge and no generic method exists. In this work, a set of simple analytical expressions is derived for the intensity distribution in convergent-beam electron diffraction (CBED) patterns recorded under three-beam conditions. It is shown that these expressions can be used to identify features in three-beam CBED patterns from which three-phase invariants can be extracted directly, without any iterative refinement processes. The octant, in which the three-phase invariant lies, can be determined simply by inspection of the indexed CBED patterns (*i.e.* the uncertainty of the phase measurement is ±22.5°). This approach is demonstrated with the experimental measurement of three-phase invariants in two simple test cases: centrosymmetric Si and non-centrosymmetric GaAs. This method may complement existing structure determination methods by providing direct measurements of three-phase invariants to replace ‘guessed’ invariants in *ab initio* phasing methods and hence provide more stringent constraints to the structure solution.

## Abstract 2

A method for measuring the three-phase invariants of crystals in any space group by inspection of convergent-beam electron diffraction patterns recorded under three-beam conditions is presented.

## 1. Introduction   

### 1.1. Tackling the phase problem with electron diffraction   

Solving a crystal structure requires knowledge of both the magnitude and phase of the structure factors. The phases are stronger constraints to a structure solution than the magnitudes (Ramachandran & Srinivasan, 1970 ▸); however, only magnitudes can be measured directly from the intensities of diffracted X-rays or neutrons (except under special experimental conditions, for example, Weckert & Hümmer, 1997 ▸). Specifically, under kinematic scattering conditions, the intensities are proportional to the modulus squared of the structure factors, so the phase cannot be measured experimentally [unless ptychography is used (Hoppe, 1969 ▸)]. This is known as the ‘phase problem’ in crystallography. The loss of phase information can often be circumvented by the application of the Patterson function (Patterson, 1934 ▸) or direct methods (Woolfson, 1971 ▸; Hauptman, 1991 ▸), provided that a sufficient number of structure-factor magnitudes are measured.

In the case of electron diffraction, the strength of the Coulombic interaction between the incident electrons and the crystal potential, means that the scattering cross section of electrons is four to five orders of magnitude larger than that of X-rays or neutrons, making dynamical scattering inevitable. Furthermore, the wavelength of the electron is small (the order of a picometre), so that the radius of the Ewald sphere is large, resulting in a high probability that the Bragg condition will be satisfied simultaneously for more than one reciprocal lattice vector. As a consequence, the intensities of diffracted electron waves depend on the phases as well as the magnitudes of the structure factors; however, the relationship is extremely complicated.

One approach to structure determination using electron diffraction is to develop techniques for mitigating the effects of dynamical scattering, so that *ab initio* phasing techniques available for kinematic diffraction data, such as direct methods (Woolfson, 1971 ▸; Hauptman, 1991 ▸) and charge flipping algorithms (Oszlányi & Sütő, 2004 ▸), can be applied with some degree of validity, although the structure solutions are usually less robust than those given by X-ray diffraction. An example of such a technique is precession electron diffraction (PED) (Vincent & Midgley, 1994 ▸; Gjønnes, 1997 ▸ ▸; Midgley & Eggeman, 2015 ▸). As for X-ray diffraction, the success of these methods improves with the number of structure-factor magnitudes that are measured and special experimental configurations, such as automated diffraction tomography (ADT)-PED (Mugnaioli *et al.*, 2009 ▸), have been devised to facilitate this. Another approach to structure determination using electrons is to embrace dynamical scattering and utilize the additional structural information it generates. If the dynamically diffracted intensities could be inverted, the phase information could be obtained through direct measurement, which can strongly confine structure solutions (Mo *et al.*, 1996 ▸; Weeks *et al.*, 2000 ▸). However, owing to the complexity of *n*-beam dynamic diffraction, no general analytical inversion (*i.e.* a mathematical description of structure-factor phases in terms of scattered intensities) under arbitrary conditions has been derived. As a commentary article has pointed out, ‘a general method of solving an unknown crystal structure with dynamic electron diffraction is yet to be developed’ (Zuo & Rouviere, 2015 ▸).

For decades, there have been ongoing efforts to develop methods that would enable the extraction of all of the structural information that is present in dynamic electron diffraction intensities. In one line of approach, structure solutions from many-beam dynamic diffraction have been pursued through numerical methods, where structure factors (both the magnitudes and the phases) can be found through optimization procedures that impose few restrictions on the initial values of structure factors (Allen *et al.*, 1998 ▸; Spence, 1998 ▸; Koch, 2008 ▸). However, the experimental implementation of these methods is challenging. So far, the retrieval of structure factors from experimental data using this class of methods has been limited to extremely thin specimens using the large-angle rocking-beam electron diffraction (LARBED) technique (Wang *et al.*, 2016 ▸).

In another line of approach, analytical inversion of three-beam dynamic electron diffraction equations has been investigated, which has resulted in the determination of three-phase invariants in centrosymmetric crystals (Moodie, 1979 ▸; Moodie *et al.*, 1996 ▸; Nakashima *et al.*, 2007 ▸, 2008 ▸, 2013 ▸). For non-centrosymmetric crystals, there have been some early attempts at analytical inversion, but these are limited to some special cases within three-beam electron diffraction, such as (i) the weak scattering case (Bird *et al.*, 1987 ▸; Bird & James, 1988 ▸), (ii) the strong coupling case (Kambe, 1957*a* ▸,*b* ▸; Zuo *et al.*, 1989 ▸) and (iii) the case where Bethe’s approximation is valid (Bethe, 1928 ▸; Zuo *et al.*, 1989 ▸). Analytical inversions, unlimited by special conditions, have so far proved impractical to implement experimentally, as they require the identification of features in the intensity distribution which cannot be easily isolated experimentally (Moodie *et al.*, 1998 ▸).

In the present work, we consider an analytical description of three-beam dynamic electron diffraction, without recourse to special conditions, which reveals a general method for the qualitative determination of three-phase invariants in both centrosymmetric and non-centrosymmetric crystals through simple inspection of convergent-beam electron diffraction (CBED) patterns.

### 1.2. A brief overview of three-beam electron diffraction   

In this work, we will derive a method for using dynamic electron diffraction at and near ‘three-beam conditions’ in CBED patterns to determine three-phase invariants in both centrosymmetric and non-centrosymmetric crystals. A three-phase invariant ϕ, is the sum of the phases φ, of three structure factors whose reciprocal lattice vectors form a closed loop, *i.e.* 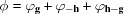. It is therefore appropriate to first review three-beam electron diffraction here.

Under three-beam diffraction conditions, the crystal is oriented with respect to the incident beam so that two, and only two, reflections simultaneously satisfy their Bragg conditions and no other reflections are excited. At and near a three-beam condition, the eigenequation for dynamic *N*-beam electron diffraction, which consists of an *N* × *N* matrix, can be approximated by an eigenequation involving a 3 × 3 matrix as follows
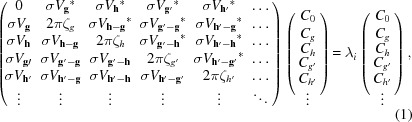
and can be approximately replaced by
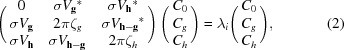
where 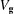 is the structure factor of the reciprocal lattice vector **g**, 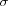 is the interaction constant, which depends on the accelerating voltage, 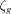 is the excitation error for reflection *g*, 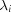 is the eigenvalue for the *i*th Bloch state (for three-beam diffraction, *i* runs from one to three), and 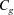 is the excitation amplitude of a certain Bloch state for reflection *g*. In equations (1) and (2), only elastically scattered electrons are considered, so the matrices are Hermitian.

Equation (2) describes the dynamic diffraction of electrons in a three-beam geometry. This is a valid approximation when two, and only two, reflections satisfy their Bragg conditions simultaneously while other beams are weakly excited. This three-beam condition can often be achieved approximately in an experiment. In the central disc of CBED or large-angle CBED (LACBED) patterns, three-beam conditions can be found at the intersections of two Bragg-condition lines [for an introduction to CBED geometry see, for example, Spence & Zuo (1992 ▸)]. Three-beam diffraction tends to be more prevalent in smaller structures rather than large ones, as in the latter case, many beams can be strongly excited simultaneously.

Now we consider situations where equation (2) holds approximately. This will result in an intensity which can be expressed in terms of only three structure-factor magnitudes (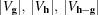), the thickness of the specimen (*z*), the incident angle (in the form of two excitation errors, 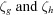) and a three-phase invariant (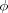). Under these circumstances, the equations describing dynamic diffraction are greatly simplified and thus, the inverse problem (describing the structure factors in terms of intensities) is made much easier.

So far, a complete inversion of the intensities which allows for experimental determination of 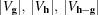 and 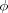 at any thickness is achievable only for centrosymmetric crystals (Moodie, 1979 ▸; Moodie *et al.*, 1996 ▸; Nakashima *et al.*, 2007 ▸, 2008 ▸, 2013 ▸), where 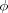 is either 0 or 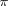. For non-centrosymmetric crystals where 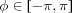, the expression for the intensities in three-beam electron diffraction is lengthy and complicated (Hurley *et al.*, 1978 ▸, 1999 ▸; Moodie *et al.*, 1998 ▸). To date, efforts to invert these equations have involved making further approximations to simplify the intensity expression so that three-phase invariants can be determined in certain special cases. For example, in the cases of weak scattering where the specimen is very thin or only weak beams are included, three-beam diffraction can be treated as a perturbation of kinematic diffraction, where a Born series including only the first- and second-order terms is used (Bird & James, 1988 ▸). However, this approximation can fail, even for specimens as thin as ∼300 Å, particularly for three-beam cases consisting of strong reflections (Guo, 2017 ▸). In practice, it is not easy to judge whether this approximation holds for a CBED pattern from an unknown crystal structure recorded at an unknown thickness, and the determination of the sign of three-phase invariants can still be ambiguous (Marthinsen, 1993 ▸). Bethe’s (1928 ▸) and Kambe’s (1957*a* ▸) formulations treated three-beam electron diffraction equations as perturbations of two-beam dynamic diffraction. However, these approximate formulations cannot distinguish the sign of 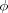 (Zuo *et al.*, 1989 ▸), even though the three-beam diffraction intensities indeed contain the sign. Therefore, none of the existing theories are applicable to general cases of three-beam electron diffraction in non-centrosymmetric crystals.

### 1.3. An outline of the current work   

The current work starts from the equation describing the intensity arising from diffraction by an arbitrary crystal potential and makes two approximations: (i) that the three-beam approximation is valid and (ii) the scattered electrons do not lose or gain energy (inelastic scattering is ignored). From two sets of reduced forms of intensity expressions, we can derive rules for determining if 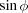 and 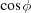 are >0, ≃0 or <0 (there are eight combinations), from different regions of the CBED patterns. Therefore, the octant in which the three-phase invariant lies can be determined, *i.e.* the uncertainty of the phase measurement is ±22.5°. We show that phase-invariants can be determined just from observations of indexed CBED patterns, without additional structural information or quantitative measurement of the thickness of the TEM specimen.

## 2. Theory   

**Figure 1:**
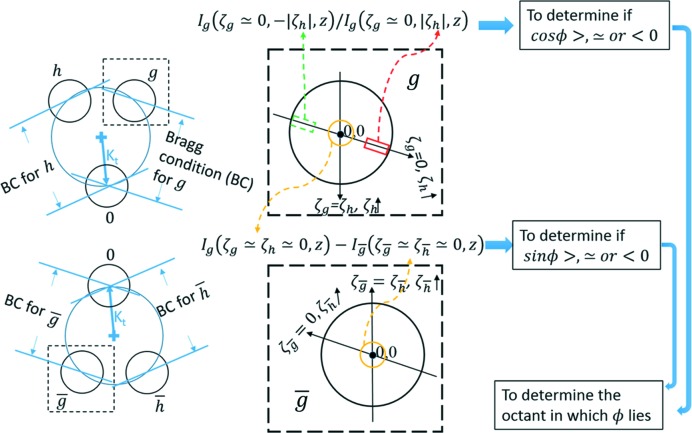
The schematic of a pair of three-beam CBED patterns that involve two Friedel pairs, *g* and 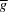, and *h* and 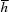, which are used to determine the octant of the three-phase invariant (the uncertainty of the phase measurement is ±22.5°). The pair of three-beam CBED patterns share the same zone axis that is perpendicular to the plane (and we define it as the ZOLZ plane) formed by the reciprocal lattice vectors **g**, **h**, −**g** and −**h**, but have different Laue circles, where the projections of the incident wavevector onto the ZOLZ plane, **K**_t_ (pointing from the centre of Laue circle to a point of interest in the central disc), are in opposite directions. In the magnified view of disc *g*, two loci, 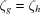 and 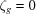 are labelled, and the intersection is the exact three-beam condition (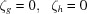). Different parts in the three-beam CBED patterns which are marked with circles and rectangles are compared in order to determine the signs of sin ϕ and cos ϕ. These, together with whether sin ϕ (or cos ϕ) is zero, can be used to constrain the three-phase invariants to within an octant (*i.e.* 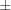 22.5°).

Commencing from the formulation derived from projection operators (Hurley *et al.*, 1978 ▸), we are able to derive the intensity expression for three-beam electron diffraction (see S1 of the supporting information). Then, we reduce the intensity expression to, 
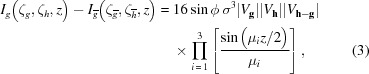
and 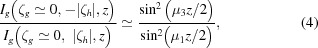
where 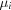 is the difference between two eigenvalues, such that 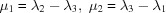, 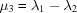. The sign convention of the three-phase invariant, 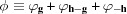, is defined in this paper such that the reciprocal lattice vectors **g**, **h**−**g** and −**h** form a closed loop in the counter-clockwise direction. The symbols ζ_*g*_ and ζ_*h*_, represent the excitation errors for reflections *g* and *h*, respectively, which together specify the angle of incidence of the electron beam with respect to the crystal. Since every point within a CBED disc corresponds to a certain angle of incidence, a coordinate system in which the two axes are 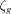 and 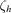 can be constructed in each disc (see Fig. 1 ▸), and the coordinate of a point can be written as (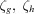). It is important to emphasize that (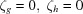) denotes the exact three-beam condition and *g* and 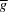 form a Friedel pair. The symbol, 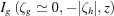, denotes the intensity of reflection *g* near its Bragg condition on the negative side of ζ_*h*_. The difference between two eigenvalues μ_*i*_, is a function of the three structure-factor magnitudes, two excitation errors and cos ϕ. As a convention in this paper, the three branches of the dispersion surface (which is a three-dimensional plot of the eigenvalue λ_*i*_ *versus* the angle of incidence; a cut view of the dispersion surfaces or dispersion curves is given in Fig. S1) are labelled as λ_1_, λ_2_ and λ_3_, such that λ_1_ > λ_2_ > λ_3_, and thus μ_1_ > 0, μ_3_ > 0 and μ_2_ = −(μ_1_ + μ_3_) < 0.

Equations (3) and (4) expose the nature of the dependence of the three-beam scattered intensity distribution on the three-phase invariant and enable the identification of simple methods to measure this, as described step by step in the following sections.

### 2.1. Qualitative measurement of sin ϕ from equation (3)   

We first consider how to measure sin ϕ from equation (3), which is identical to equation (23) in the work by Hurley *et al.* (1999 ▸) except for the right-hand side which is factorized. The left-hand side represents the intensity difference between a Friedel pair of reflections, *g* and 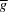 (which can also be *h* and 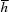), at the same thickness. Two Friedel pairs, one with *g* and 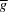 and the other with *h* and 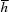, form two separate three-beam conditions, 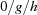 and 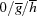. If the zone axis is set perpendicular to the plane formed by the reciprocal vectors **g** and **h** (which is also formed by −**g** and −**h**), the centres of the Laue circles for the three-beam conditions 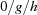 and 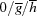 are on the opposite side of each other (see Fig. 1 ▸). The two three-beam conditions are typically tens of mrad (or equivalently ∼0.5 Å^−1^ at 200 kV) apart. To achieve this experimentally, two CBED patterns from different incident angles or a single large-angle CBED pattern are/is needed. The right-hand side of equation (3) is the product of three structure-factor magnitudes, sin ϕ, and the product of three thickness-dependent terms. The benefit of factorizing the right-hand side is to determine the maximum thickness, below which, the sign of the intensity difference depends only on the sign of sin ϕ, *i.e.* when 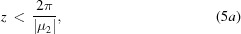
we have 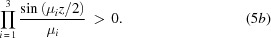
In the vicinity of the three-beam conditions, (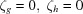), inequality (5*a*) can be satisfied for a broad range of specimen thicknesses, which makes the measurement feasible. In this case,
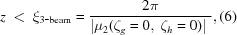
where we define ξ_3-beam_ as the ‘three-beam extinction distance’, that is the thickness *z* = ξ_3-beam_ when the intensity difference at the three-beam condition vanishes. The range of thicknesses that satisfy the inequality (6) is very large in many cases (to gain an impression of typical values of the three-beam extinction distances, see Appendix *A* [App appa]). Nevertheless, we have derived a rule (which will be stated in the following section) for checking if the inequality (6) is satisfied for an unknown thickness.

Instead of comparing a Friedel pair from two CBED patterns, we can compare the two diffracted beams within a single three-beam CBED pattern if they have the same structure-factor magnitudes, for example, if they form a Bijvoet pair such as *hkl* and *h* 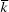 *l*. In this case, only a single three-beam CBED pattern is needed to determine the sign of sin ϕ. If sin ϕ = 0, Friedel’s law will be preserved for all thicknesses according to equation (3).

### 2.2. Qualitative measurement of cos ϕ from equation (4)   

Equation (4) is formulated to enable the practical identification of the sign of cos ϕ. Equation (4) holds only approximately for the regions that are close to the two-beam condition, however, away from the three-beam condition (as discussed in section S1.3 of the supporting information), the approximation is valid for the purpose of determining the sign of cos ϕ.

By solving equation (2) for λ_*i*_ (which gives a cubic equation), it can be proven analytically that the sign of cos ϕ has a direct dependence on the relative magnitudes of μ_3_(−|ζ_*h*_|) and μ_1_(|ζ_*h*_|), which are on opposite sides of the three-beam condition in each CBED disc, such that 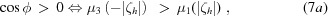
and 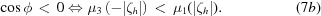
Therefore, for a specimen that is not very thick, *i.e.* 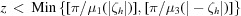, one can decide the sign of cos ϕ by direct comparison of the intensities between the negative and the positive sides of ζ_*h*_ within reflection *g* or 0 (Figs. 1 ▸ or 5). For a thicker specimen, we are also able to determine the sign of cos ϕ by comparing the same two regions as mentioned above: the factorized form of 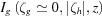 has a thickness-dependent factor of {[sin^2^(μ_*i*_ *z*/2)]/μ_*i*_^2^}, which is similar in form to the two-beam intensity expression. Therefore, in the regions where equation (4) is valid, oscillating ‘thickness fringes’ can be observed and the spacings of the thickness fringes are different on different sides of the three-beam condition. The difference in the spacing depends on the relative magnitudes of μ_3_(−|ζ_*h*_|) and μ_1_(|ζ_*h*_|), and thus depends on the sign of cos ϕ.

When cos ϕ = 0, a centre of inversion is present in the three-beam condition at all thicknesses.

### 2.3. Determination of ϕ   

Once we have determined if sin ϕ and cos ϕ are positive, negative or approximately zero, we are then able to determine the octant in which the three-phase invariant ϕ lies (*i.e.* the uncertainty is ±22.5°) according to Table 1 ▸.

## 3. Practical procedures for determining three-phase invariants (ϕ)   

In the previous section, we explained the theoretical basis for the phase determination with equations and schematics. These theories lead to simple criteria for determining the signs of sin ϕ and cos ϕ (and also whether they are zero) by qualitative observations of the CBED patterns. In particular, these signs can be determined without the need for quantitative measurement of diffracted intensities or numerical simulation. In this section, we state these criteria explicitly and illustrate with examples using simulated CBED patterns in three-beam orientations. These criteria are collated into a flowchart which provides step-by-step instructions for using three-beam CBED to determine three-phase invariants by inspection (the flowchart is shown in Fig. 7 ▸ after summarizing all of the criteria). In order to emulate realistic experimental data, the simulations of CBED patterns were performed using *JEMS* (Stadelmann, 2004 ▸) using the Bloch wave formalism, incorporating more than 100 beams and including absorptive potentials to model the effect of inelastic scattering (*i.e.* the approximations used in deriving the analytical expressions were not employed in the simulations). For practical purposes, users of the present three-beam method can refer to the current section alone without concerning themselves with the mathematics in the previous section.

From equation (3), we can directly establish the following criterion.

**Criterion 1. To decide whether |*V_h−g_*| sin ϕ = 0**

At any thickness, if 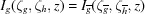, *i.e.* Friedel’s law is preserved, then 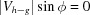.

To determine the sign of sin ϕ by inspection only, it needs to be ensured that the thickness cannot exceed a certain value, the so-called three-beam extinction distance ξ_3-beam_.

**Criterion 2. To decide if *z* < ξ_3-beam_**

When both of the following conditions are satisfied, then *z* < ξ_3-beam_.

**Figure 2:**
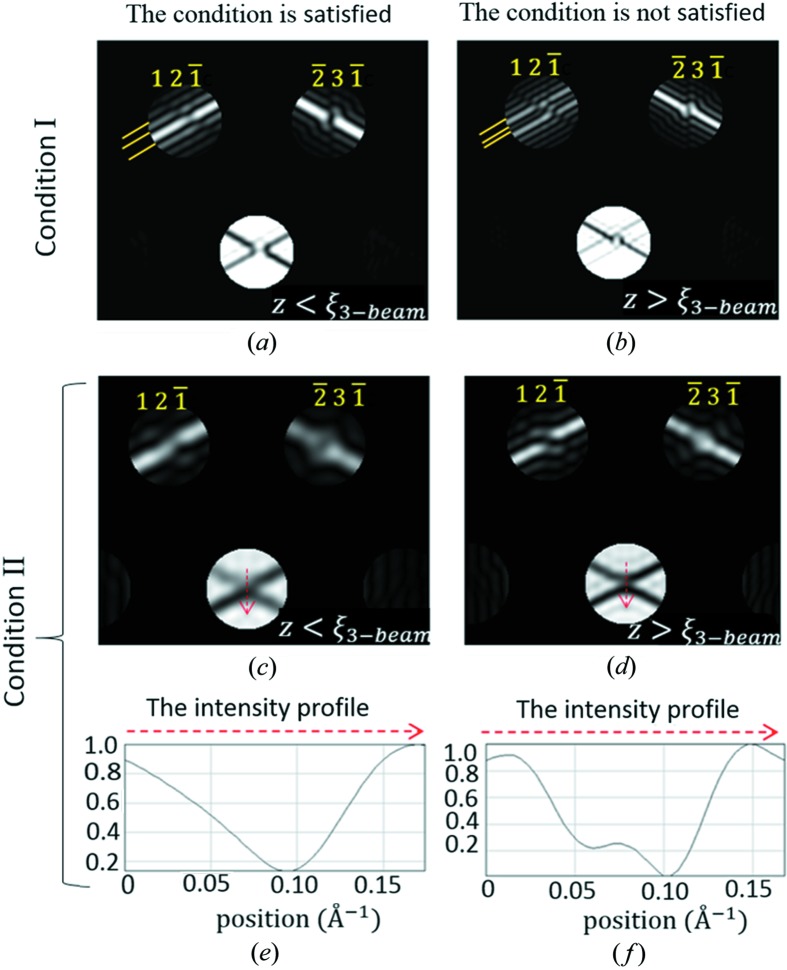
Illustration of *Criterion 2* [Statement enun2] with simulated CBED patterns from alpha-quartz (space group *P*3_1_21) near [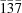] at 200 kV. Condition I is satsified in (*a*) but not in (*b*). The yellow lines label the width of the first bright fringe relative to the second one. Condition II is satisfied in (*c*) but not in (*d*). The intensity profiles along the dashed red arrows in (*c*) and (*d*) are shown in (*e*) and (*f*), respectively. In the calculation of (*c*) and (*d*), the same set of parameters as those in the calculation of (*a*) and (*b*) were used except that the coupling structure factor, 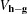, was artifically increased to produce a strong coupling case of three-beam diffraction as opposed to the weak/moderate coupling case in (*a*) and (*b*).

*Condition I*. In disc 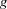, the excess Bragg line away from the three-beam condition has a central bright fringe that is more than 1.6× wider than the neighbouring bright fringes (Fig. 2 ▸ *a*).

*Condition II*. In disc 0, the intensity profile along the locus 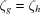 has no maximum but a local minimum at or near the exact three-beam condition, *i.e.* 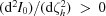 (Figs. 2 ▸ *c* and 2*e*).

The detailed derivations of *Criterion 2* [Statement enun2] can be found in section S2 of the supporting information.

By comparing a Friedel pair of reflections, *g* and 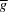, from the couple of three-beam conditions that consist of two Friedel pairs, 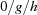 and 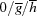, the sign of 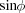 can be determined as follows.

**Criterion 3. To determine the sign of sin ϕ**

**Figure 3:**
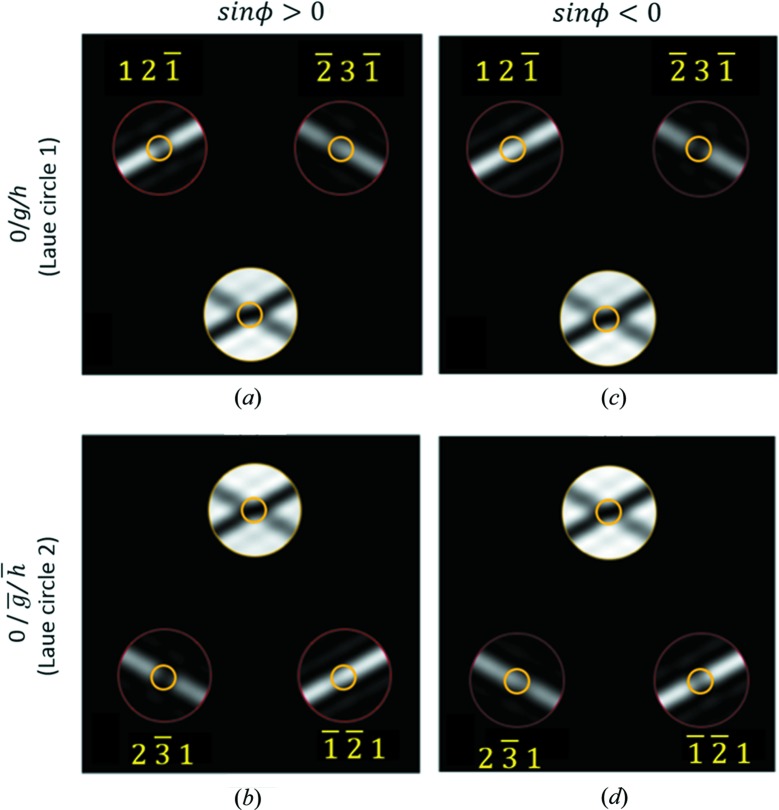
An illustration of *Criterion 3* [Statement enun3] using simulated CBED patterns of alpha-quartz near [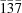]. Two different types of alpha-quartz which belong to different space groups are used: the structure in (*a*) and (*b*) is in the space group *P*3_2_21, the structure in (*c*) and (*d*) is in the space group *P*3_1_21. The two structures are enantiomorphs of each other. The yellow circles enclose the three-beam conditions and their neighourhoods. For the structure in (*a*) and (*b*), 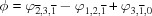 = 103°. For the structure in (*c*) and (*d*), 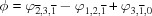 = −103°.

If *z* < ξ_3-beam_, and 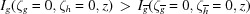, then 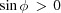 (Figs. 3 ▸ *a*, 3*b* and 4 ▸ *a*); if *z* < ξ_3-beam_, and 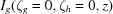 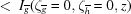, then 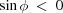 (Figs. 3 ▸ *c*, 3*d* and 4 ▸ *b*).

**Figure 4:**
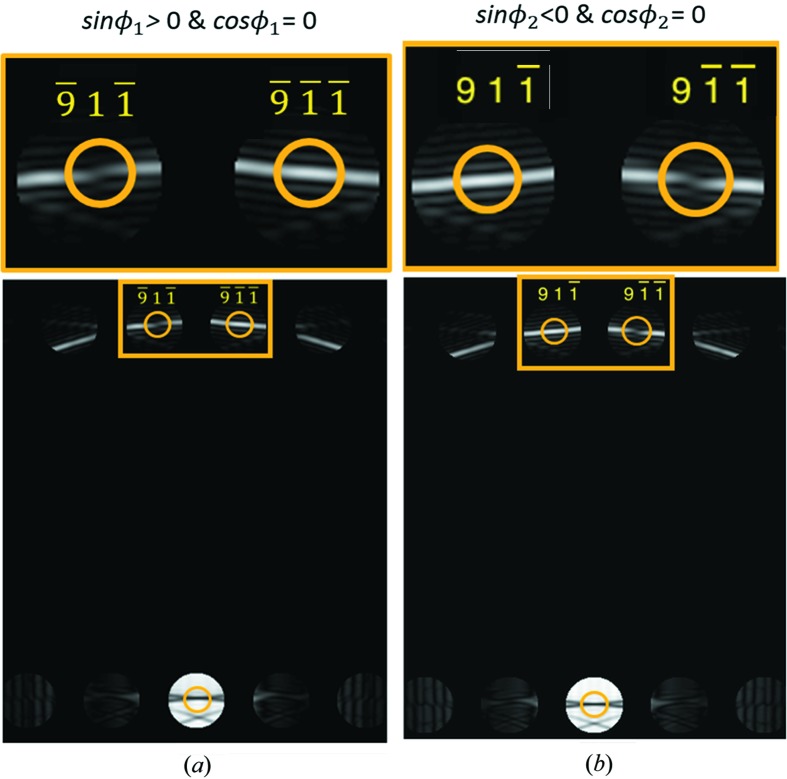
Illustrations of *Criteria 3* [Statement enun3] and *4* [Statement enun4] in CBED patterns with Bijvoet pairs satisfying the three-beam conditions. The CBED patterns were simulated for GaAs in the zone axes (*a*) [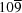] and (*b*) [109]. The three-beam conditions of interest are circled. An enlarged view of reflections *g* and *h* (yellow boxed region) is also shown. Note that 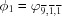 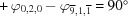 and 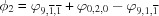 = −90°.

If a Bijvoet pair exists, we can also apply *Criterion 3* [Statement enun3] to determine the sign of sin ϕ, with the exception of replacing 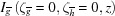 with 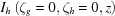, see examples in Fig. 4 ▸.

Now we come to the criteria for the determination of cos ϕ.

**Criterion 4. To determine if |*V_h−g_*| cos ϕ = 0**

At any thickness, if 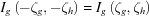, *i.e.* a centre of inversion is present in the three-beam condition, then 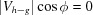 (Fig. 4 ▸). Otherwise, 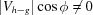.

**Figure 5:**
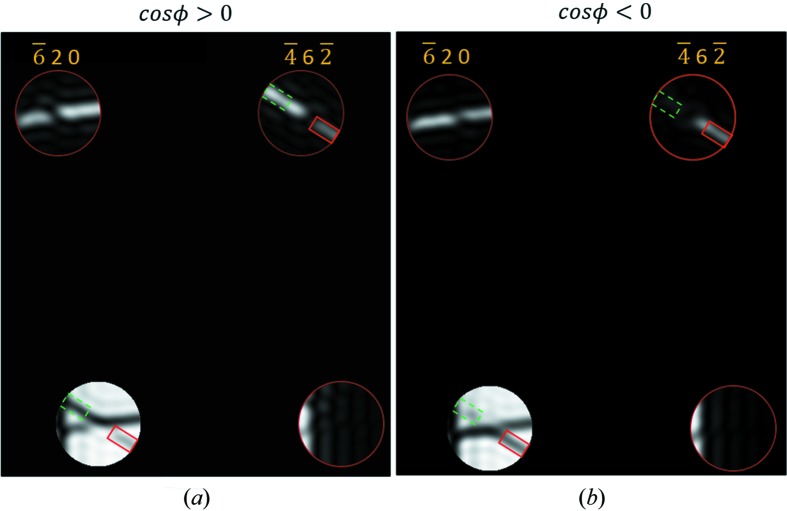
Illustrations of *Criterion 5* [Statement enun5] for determining the sign of cos ϕ for thin specimens. The examples are simulated CBED patterns from (*a*) real and (*b*) artificial structures of ZnS in a [137] zone axis orientation. An approximate three-beam condition is located in the centre of each disc. Following the convention in Fig. 1 ▸, the regions with negative and positive ζ_*h*_ are labelled with equally sized rectangles with dashed (green) and solid (red) lines, respectively. In order to facilitate an inspection, the rectangles are located away from the exact three-beam condition by the same distance. In the simulations, the following values for the three-phase invariant and the thickness were used: (*a*) 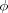 = 2.5°, *z* = 700 Å; (*b*) 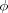 = −140°, *z* = 700 Å. In the calculation of (*a*), the three-phase invariant was adopted from the structure itself whereas in the calculations of (*b*), the three-phase invariant was artificially adjusted in order to produce a situation such that cos ϕ < 0. In this adjustment, the phase of 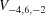 is changed to −140° and the phase of its complex conjugate, which is equivalent to 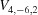, is changed to +140° simultaneously.

By comparing the two regions, 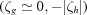 and (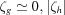), labelled as rectangles with dashed (green) and solid lines (red) in both Figs. 5 ▸ and 6 ▸, one can determine the sign of 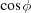 for both thin and thick specimens by checking Figs. 5 ▸ and 6, ▸ respectively.

Criterion 5. To determine the sign of 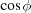 for thin specimens  

*I_g_*(− |ζ_*h*_|) > *I_g_*(|ζ_*h*_|) or *I*_0_(− |ζ_*h*_|) < *I*_0_(|ζ_*h*_|) ↠ cos ϕ > 0 (Fig. 5*a* ▸)

*I_g_*(− |ζ_*h*_|) < *I_g_*(|ζ_*h*_|) or *I*_0_(− |ζ_*h*_|) > *I*_0_(|ζ_*h*_|) ↠ cos ϕ < 0 (Fig. 5*b* ▸)

**Criterion 6. To determine the sign of cos ϕ for thick specimens**

The thickness fringes, which are located near the Bragg condition lines for 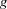 but away from the three-beam condition in disc 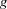, are inspected.

**Figure 6:**
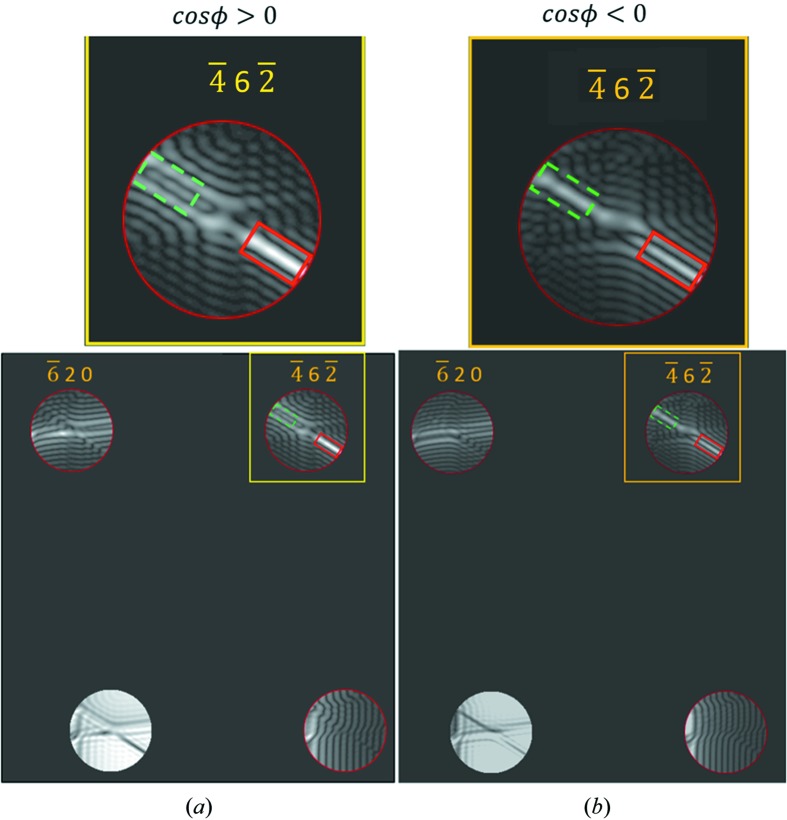
Illustrations of *Criterion 6* [Statement enun6] for determining the sign of cos ϕ for thick specimens. The examples are simulated CBED patterns from ZnS [137] near a three-beam condition which is located in the centre of each disc. Following the convention in Fig. 1 ▸, the regions with negative and positive ζ_*h*_ are labelled with equally sized rectangles with dashed (green) and solid (red), respectively. In order to facilitate an inspection, the rectangles are located away from the exact three-beam condition by the same distance. In the simulations, the following values of the three-phase invariant and the thickness were used: (*a*) ϕ = 2.5°, *z* = 1720 Å; (*b*) ϕ = −140°, *z* = 1980 Å. In the calculation of (*a*), the three-phase invariant was adopted from the structure itself whereas in the calculations of (*b*), the three-phase invariant was artificially adjusted in order to produce a situation such that cos ϕ < 0. The same adjustment as in Fig. 5 ▸ has been performed.

If the fringe spacing on the negative side is less than that on the positive side, then 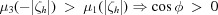 (Fig. 6 ▸ *a*), otherwise 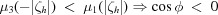 (Fig. 6 ▸ *b*).

**Figure 7:**
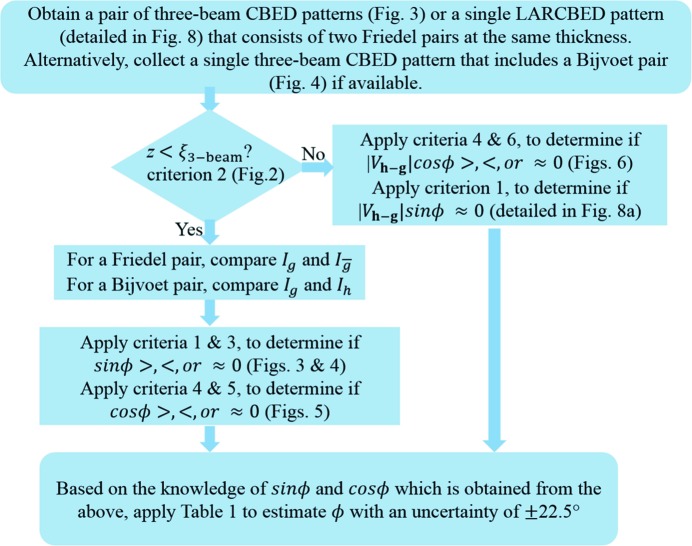
A flowchart that summarizes the procedures for determining the three-phase invariants from three-beam CBED patterns.

*Criterion 6* [Statement enun6] can be applied to thick specimens, typically up to 2000 Å thick for most inorganic crystals (2000 Å is generally much thicker than most TEM specimens of inorganic crystals). The six criteria are assembled into a flowchart in Fig. 7 ▸ which summarizes the procedure for determining three-phase invariants.

## 4. Experimental demonstration   

In this section, we demonstrate the procedures in Section 3, with experimental data as a proof of concept. Electron diffraction experiments are carried out on a centrosymmetric crystal (Si) and a non-centrosymmetric crystal (GaAs) to demonstrate the feasibility of direct measurement of three-phase invariants.

The experiments were conducted on a JEOL 2100F transmission electron microscope (TEM) and the patterns were recorded on a Gatan Ultrascan 1000 CCD camera. Commercial software, *QED* (HREM Research Inc., 2012 ▸) as a plug-in for *DigitalMicrograph* (Gatan Inc., Pleasanton, CA), was used to generate large-angle rocking-convergent-beam electron diffraction (LARCBED) patterns to measure the three-phase invariants, as the large angular range increases the opportunities for capturing several different three-beam conditions in a single pattern. LARCBED is similar to large-angle rocking-beam electron diffraction (LARBED) (Koch, 2011 ▸) except that convergent illumination is used instead of parallel illumination. In LARCBED, the incident beam is tilted about a spot on the specimen and CBED patterns are recorded sequentially over a grid of different beam tilts. Through cutting and stitching CBED discs for each reflection from the patterns recorded at all of the beam tilts, a large-angle CBED pattern can be reconstructed. An analogous approach for achieving large-angle CBED patterns from nano-sized areas has also been demonstrated by Beanland *et al.* (2013 ▸). Unlike LACBED (Tanaka *et al.*, 1980 ▸), where the probe is not focused in the specimen plane, a reconstructed LARCBED pattern can be obtained from a smaller specimen volume because all the CBED patterns that contribute to the LARCBED pattern are obtained from a probe focused on the same position on the specimen. The tilt-induced beam shift is compensated for by the application of *QED* software to keep the probe on the same specimen area. Nevertheless, there is still a residual beam shift, the size of which depends on the electron-optics of a given microscope and was approximately 10 nm in the present case. The large angular range provided by these LARCBED patterns significantly improves the likelihood of satisfying the three-beam conditions. Usually, a LARCBED pattern can cover several three-beam diffraction conditions, among which, a pair of three-beam conditions that involve two Friedel pairs (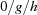 and 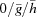) can be found. This allows for the comparison of a Friedel pair satisfying three-beam conditions that are tens of mrad (equivalent to ∼0.5 Å^−1^ at 200 kV) apart at the same thickness, which makes it experimentally feasible to apply *Criteria 1* [Statement enun1] and *3* [Statement enun3].

### 4.1. The experimental procedures   

First, standard alignments for CBED were performed using a convergence angle that avoided overlap between adjacent discs. Then, the deflection system was calibrated and the aberration-induced beam shifts were compensated for in CBED mode by using *QED* software to minimize beam shift on the specimen as the beam was tilted. Third, the data collection was initiated by *QED*. When the data collection was finished, a LARCBED pattern could be reconstructed from the set of CBED patterns.

**Figure 8:**
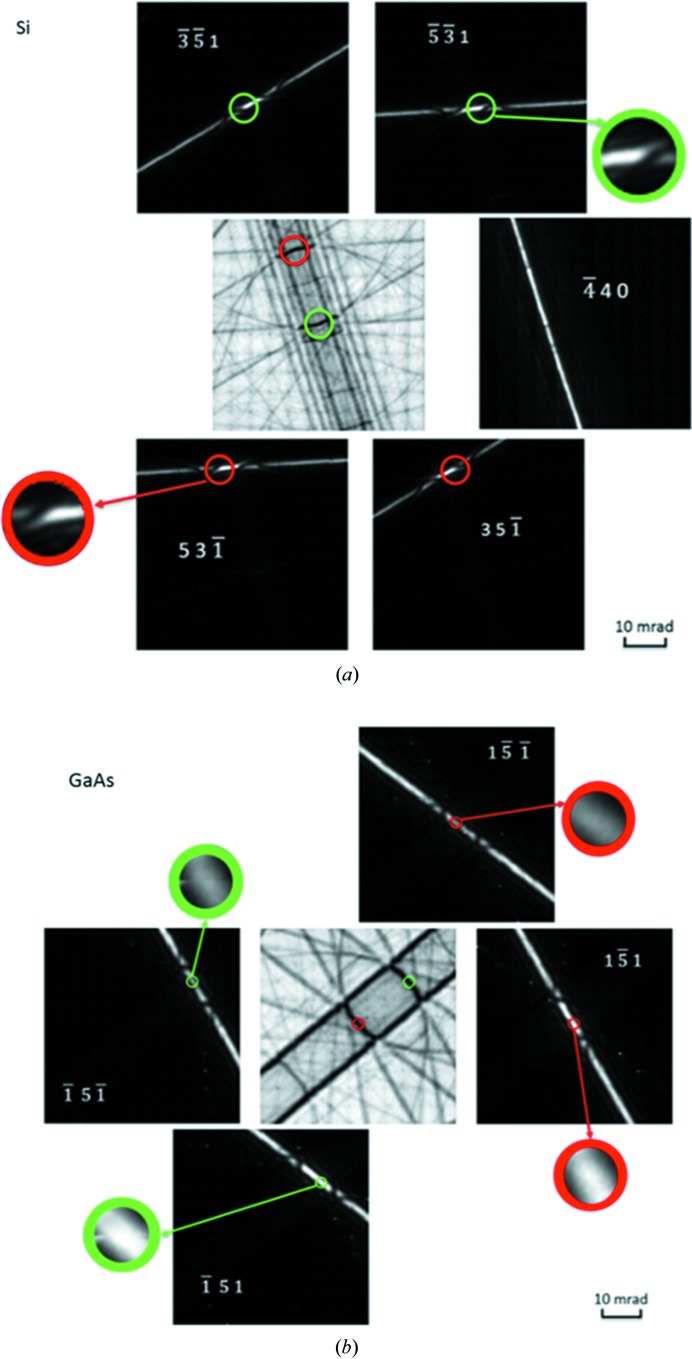
Reconstructed LARCBED patterns of a few selected reflections for (*a*) Si [118] and (*b*) GaAs [510] at 200 kV. In each figure, a pair of three-beam conditions that involve two Friedel-pair reflections are labelled with circles in red (dark in greyscale) and green (light in greyscale). The sizes of the circles are chosen such that the three-beam conditions and their closest neighbourhoods are included while the neighbouring Bragg conditions are avoided.

The LARCBED patterns in Figs. 8 ▸(*a*) and 8(*b*) were reconstructed from 121 CBED patterns recorded from Si near the [118] zone axis and GaAs near the [510] zone axis, respectively.

### 4.2. Application of the reconstructed LARCBED patterns   

The reconstructed central beam is useful for searching for three-beam conditions, which can be found at the intersection of lines defining two Bragg conditions (usually two dark lines at low thicknesses) where no other Bragg condition lines for strong reflections lie in the neighbourhood. Then, the corresponding three-beam conditions in the diffracted beams can be located as they lie in the same position as in the central beam. For example, in Fig. 8 ▸(*a*), a pair of three-beam conditions (including the neighbourhoods), 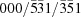 and 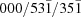, are labelled in the reconstructed LARCBED pattern with green and red circles, respectively. All the three green circles enclose the same three-beam condition for 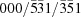 and its neighbourhood. In the same place in the other diffracted beams such as reflection 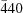, the diffraction pattern is comparatively dark and featureless, simply because the three-beam interaction amongst 000, 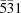 and 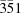 dominates in this orientation.

### 4.3. Practical examples of determining the three-phase invariants   

Once we have circled the regions of three-beam conditions, we can proceed to determine the three-phase invariants following the flowchart in Fig. 7 ▸.

#### 4.3.1. Example 1, Si   

First, consider the experimental LARCBED pattern from Si (Fig. 8 ▸ *a*) as an example.

(i) According to *Criterion 2* [Statement enun2], the thickness of the specimen satisfies the condition, *z* < ξ_3-beam_, which means we are allowed to determine the sign of the three-phase invariant from inspection.

(ii) By comparing the Friedel-pair reflections, 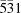 and 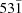, there is centrosymmetry/twofold symmetry between the intensity distributions within the green circle in the 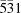 disc and within the red circle in the 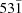 disc. According to *Criterion 1* [Statement enun1], 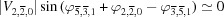.

(iii) According to *Criterion 4* [Statement enun4], 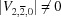, and thus 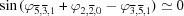.

(iv) According to *Criterion 5* [Statement enun5], intensity asym­metry about the three-beam conditions reveals 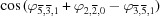 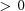.

(v) From Table 1 ▸ we can conclude that 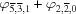 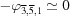. This agrees with the known value for Si of 0°.

#### 4.3.2. Example 2, GaAs   

Here we take the experimental LARCBED pattern from GaAs (Fig. 8 ▸ *b*), as a non-centrosymmetric example.

(i) According to *Criterion 2* [Statement enun2], the thickness of the specimen satisfies the condition z < ξ_3-beam_.

(ii) By comparing the Friedel-pair reflections 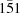 and 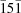, we can see that 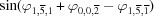 > 0 from the intensity difference according to *Criterion 3* [Statement enun3].

(iii) According to *Criterion 4* [Statement enun4], since there is a centre of inversion at the exact three-beam condition, 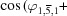 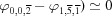.

(iv) According to Table 1 ▸, 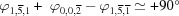. The result of this qualitative measurement agrees approximately with the known value for GaAs of +88°.

## 5. Potential applications   

### 5.1. Improvement in *ab initio* phasing   

If the ‘guessed’ three-phase invariants in direct methods (Woolfson, 1971 ▸; Hauptman, 1991 ▸), which are based on probability, are replaced by three-phase invariants measured directly from three-beam diffraction, then the success rate and accuracy of *ab initio* phasing can be greatly improved: a small set of measured three-phase invariants with a mean error of ± 22.5° (Mo *et al.*, 1996 ▸) (*i.e.* which is equivalent to finding the octant of the three-phase invariants as achieved in this article) or even 40° (Weeks *et al.*, 2000 ▸) may enable a structure solution where it would otherwise be impossible. In such cases, fewer structure-factor magnitudes would need to be measured.

Therefore, one application of the current three-beam method is to combine it with X-ray diffraction data or with electron-diffraction data based on quasi-kinematic diffraction such as PED (Vincent & Midgley, 1994 ▸) to improve *ab initio* phasing. Importantly, combining this three-beam method with PED may help to solve structures for nano-sized crystals which can be most readily studied using the <1 nm probes that are routinely available in TEMs.

### 5.2. Resolution of enantiomorphs   

For a pair of enantiomorphically related structures (*L* and *R*), the same three-phase invariants have opposite signs, *i.e.* 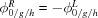, and the three-phase invariants for the opposite three-beam conditions have the same sign, *i.e.* 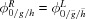. Therefore, to identify the chirality of a structure, both the signs of the indices (of reflections *g* and *h*) and the sign of the three-phase invariant, 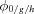, have to be determined consistently. Since three-beam diffraction alone involves three reciprocal lattice vectors that are coplanar, it is impossible to identify chirality, which is three-dimensional, unless a fourth beam that is not in the same zone axis (non-coplanar) is present in the field of view (Spence *et al.*, 1994 ▸). Therefore, at least one three-beam condition and one HOLZ reflection should be present in the same diffraction pattern.

Within the bright-field component of a reconstructed LARCBED pattern, a pair of three-beam conditions that involve two Friedel pairs and deficit HOLZ lines can be found simultaneously. Based on the positions of the deficit HOLZ lines relative to the three-beam conditions in the bright field, a Friedel pair can be indexed without ambiguity. The sign of three-phase invariants can be determined from direct observations following the present method. Therefore, chirality can be clearly identified from just a single LARCBED pattern (with both the central and diffracted beams) where a Friedel or Bijvoet pair of reflections satisfies three-beam conditions.

## 6. Challenges and limitations   

### 6.1. Applicability to large structures   

The three-beam approximation in equation (2) can hold very well if the excitation errors of the reflections other than reflections *g* and *h* are much larger than any structure-factor magnitude, *i.e.*

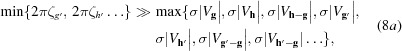
and thus,
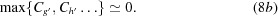
It is easy to find three-beam diffraction conditions in structures with small unit cells (say, with cell volumes less than a few thousand Å^3^) because their reciprocal lattice points are sparsely spaced and reflections other than *g* and *h* tend to be weakly excited near the three-beam condition for 0/*g*/*h*. In contrast, the projection of the reciprocal lattice for large structures is dense and other reflections *g*′, *h*′…, tend to be strongly excited at the same time (their excitation errors, 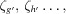 tend to be small). Three-beam conditions can be difficult to isolate in large structures and the influence of many-beam diffraction effects is difficult to avoid. Nevertheless, if the structure-factor magnitudes relevant to a particular three-beam diffraction condition, 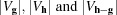, are much larger than those of the other excited reflections (like 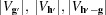, *etc*.), then many-beam diffraction can still be treated as a perturbation of the three-beam approximation. This has been shown by studies in three-beam X-ray diffraction (Weckert & Hümmer, 1997 ▸). Three-beam diffraction of X-rays has been demonstrated in some small protein crystals such as myoglobin (Hümmer *et al.*, 1991 ▸), tetragonal lysozyme and catalase oxidoreductase (Weckert *et al.*, 1993 ▸).

Since the Ewald sphere for high-energy electron diffraction is much ‘flatter’ than that of soft X-ray diffraction, which results in many more reflections being excited simultaneously, the applicability of three-beam electron diffraction to large structures like protein crystals may be very limited. Nevertheless, the applicability of three-beam electron diffraction to moderately complex structures (a unit cell with dozens to a few hundred atoms) can be expected. Feasibility may be increased with the use of the new generation of very low voltage TEMs (>30 kV), offering large electron wavelengths and hence increased curvature in the Ewald sphere.

### 6.2. Applicability to beam-sensitive structures   

Under standard operating conditions, with no effort to minimize electron dose, the total dose can be large and unsuitable for beam-sensitive structures. For example, the set of 121 CBED patterns at 200 kV (which gave the LARCBED patterns in Fig. 8 ▸) was estimated to use a total dose of 10^5^ e Å^−2^. However, it is important to note that the three-beam method described here relies only on a qualitative inspection of features in the CBED patterns, rather than quantitative measurement of absolute intensities. It can therefore tolerate a high noise level in the diffraction data, so that low-dose CBED methods, such as those used by Wu & Spence (2003 ▸), can be used. Coupled with a new generation of high-sensitivity and fast detectors, low-dose three-beam measurements of three-phase invariants in beam-sensitive materials is likely to be very feasible.

## 7. Clarifying points   

### 7.1. Crystallographic phase *versus* phase of the exiting electron wave   

The current three-beam method provides direct measurement of three-phase invariants (which is the phase information of structure factors) rather than the phase of exiting electron waves, which can be measured by electron holography (Gabor, 1948 ▸), through focal series methods (Schiske, 1968 ▸, 2002 ▸; Kirkland, 1984 ▸) or electron ptychography (Rodenburg, 2008 ▸; Humphry *et al.*, 2012 ▸). Under dynamical scattering conditions, there is no direct or general analytical relationship between the phase of the exit wavefunction and the phase of the structure factors.

### 7.2. Comparison with electron ptychography for structure-factor phase determination   

Apart from the current three-beam CBED approach, electron ptychography can also provide a measurement of structure-factor phases (Nellist *et al.*, 1995 ▸) and thus, three-phase invariants, provided the single scattering condition prevails. Therefore, it requires extremely thin and weakly scattering specimens. Electron ptychography uses the coherent interference in overlapping CBED discs and hence requires an effective source that is spatially coherent over the angular range of the disc and a probe-forming lens system that does not introduce significant coherent aberrations within this range. In contrast, three-beam CBED is based on dynamical scattering and is thus valid for thicker specimens. Furthermore, as the discs do not overlap, it is independent of coherent aberrations [such an independence can be simply proven by deriving the dynamical intensities in CBED without coherent interference, from the exit wavefunction which contains the initial phase of the probe, such as equation 14.87 in the book by Zuo & Spence (2017 ▸); for an earlier reference, see Spence & Cowley (1978 ▸)].

### 7.3. Comparison with quantitative CBED   

The current three-beam CBED method is an *ab initio* approach where no structural model is assumed. This is in contrast to quantitative CBED for refining structure factors and three-phase invariants within a given structural model (Goodman & Lehmpfuhl, 1967 ▸; Spence, 1993 ▸; Nakashima, 2017 ▸). A quantitative analysis of experimental three-beam CBED patterns has been used to refine three-phase invariants to an accuracy of within one degree (for example, Høier *et al.*, 1999 ▸). However, the quality of an experimental pattern for quantitative CBED needs to be much higher than required by the current method, where only qualitative inspection is involved.

## 8. Conclusions   

This article has introduced an *ab initio* method using three-beam convergent-beam electron diffraction (CBED) for the practical determination of three-phase invariants in non-centrosymmetric as well as centrosymmetric crystals. It can readily be applied to nano-sized crystals. Starting from the exact solutions to three-beam electron diffraction, we have derived a theory which allows for the inversion of the diffracted intensities to determine the signs of sin ϕ and cos ϕ (and also whether they are close to zero). Based on this theory, we have provided instructions for determining the octant of three-phase invariants (*i.e.* the uncertainty in the determination of three-phase invariants is ±22.5°) by qualitative inspection of indexed CBED or LARCBED patterns.

Importantly, only qualitative inspection of the diffraction patterns is required, without any need for quantitative intensity measurement or numerical pattern matching or refinement. No additional knowledge about the structure or the specimen thickness, is needed (except for the pattern indices).

LARCBED experiments on a centrosymmetric crystal of Si and a non-centrosymmetric crystal of GaAs have been carried out to demonstrate the current method.

Three-beam CBED can be combined with X-ray and precession electron diffraction data to improve *ab initio* phasing. Also, the enantiomorph ambiguity can be eliminated by observations of the LARCBED patterns where HOLZ reflections and three-beam conditions are present in the same pattern. Furthermore, the three-beam method may have the potential to measure three-phase invariants in beam-sensitive structures using the latest generation electron detectors with high speed and sensitivity.

*Note added in proof*. It is with great sadness that we learned of the passing of Professor Alexander Moodie FAA on 8 July 2018, a pioneer in the field of electron crystallography. Among his many distinguished contributions, is the unique analytical inversion of three-beam electron-scattering equations. We dedicate this work to a brilliant scientist, inspiring colleague and generous mentor.

## 9. Related literature   

The following references are cited in the supporting information: Blackman (1939 ▸); Wolfram Research, Inc. (2014 ▸).

## Supplementary Material

Derivations for equations (3) and (4), and Criterion 2. DOI: 10.1107/S2052252518012216/gq5009sup1.pdf

## Figures and Tables

**Table 1:** Table for determining the three-phase invariants

sin ϕ	cos ϕ	Estimated ϕ (°)(with an error of ±22.5°)
≃0	>0	0
>0	>0	45
>0	≃0	90
>0	<0	135
≃0	<0	180
<0	<0	−135
<0	≃0	−90
<0	>0	−45

## Appendix A Typical values for the three-beam extinction distance, ξ_3-beam_

Here we show that the validity of the condition, *z* < ξ_3-beam_, is not restricted to very thin specimens. The three-beam extinction distance, ξ_3-beam_, decreases with increasing structure-factor magnitudes, so the range of the thickness that satisfies the condition, *z* < ξ_3-beam_, becomes narrower with larger structure-factor magnitudes. One may think that the specimen needs to be very thin for a three-beam case with large structure factors. However, it can be shown that even if the three-beam case involves large structure factors, the three-beam extinction distance is still large. For example, a three-beam case,  , in zinc blende ZnTe, where  = 4.2 V and  = 2.5 V, may serve as an example of three-beam cases with three fairly large structure-factor magnitudes since a fairly heavy element (Te) is present in the structure, and  and  are among the largest structure-factor magnitudes in this structure. In this case, the three-beam extinction distance, ξ_3-beam_, at 200 kV is about 650 Å, which is sufficiently large to allow specimen thicknesses less than ξ_3-beam_ to be accessed practically in TEM. In general, a typical value of the three-beam extinction distance, ξ_3-beam_, at 200 kV for inorganic crystal structures is above 1000 Å and for organic crystal structures it is above 2000 Å.

This relatively large range of validity may seem surprising in the context of previous methods based on the special weak scattering case [(Bird *et al.*, 1987 ▸), see comment by Marthinsen (1993 ▸)]. However, the present general three-beam method is not restricted to weak scattering, ensuring it has validity over a much wider range of thicknesses. To get a sense of the difference in the validity ranges between the kinematic approximation and current approach, the *Mathematica* code from the website https://github.com/DrYGuo/3-beam-project can be used.
