# Supplementary material for: Three-beam convergent-beam electron diffraction for measuring crystallographic phases
Source: IUCrJ. 2018 Oct 8;5(Pt 6):753–64. doi: 10.1107/S2052252518012216 (PMC6211523; doi:10.1107/S2052252518012216)
Supplement: Supplementary file 1 [file m-05-00753-sup1.pdf]

IUCrJ

Volume 5 (2018)

Supporting information for article:

Three-beam convergent-beam electron diffraction for measuring crystallographic phases

Yueming Guo, Philip Nakashima and Joanne Etheridge

Three-beam convergent-beam electron diffraction for measuring crystallographic phases

Authors

Yueming Guo[a], Philip Nakashima[a] and Joanne Etheridge[ab]*

[a]Department of Materials Science and Engineering, Monash University, Victoria, 3800, Australia

[b]Monash Centre for Electron Microscopy, Monash University, Victoria, 3800, Australia

Correspondence email: joanne.etheridge@monash.edu

S1. Derivation of equations (3) & (4)

S1.1. Derivation of a closed-form expression for three-beam intensity

Here, we derive a closed-form solution to three-beam dynamic diffraction.

The wave function of the diffracted beam g and the central beam 0 can be expressed as:

$$\psi_g = \langle g|exp(i\,\boldsymbol{M}\,z)|0\rangle \tag{S1a}$$

and

$$\psi_0 = \langle 0|exp(i\,\boldsymbol{M}\,z)|0\rangle\,, \tag{S1b}$$

where z is the specimen thickness,

$|0\rangle = (1, 0, 0)^{\mathrm{T}}$ and $\langle g| = (0, 1, 0)$

are the initial and final states of the scattering processes. The Hermitian matrix,

$$\boldsymbol{M} = \begin{pmatrix} 0 & \sigma V_{\mathbf{g}}{}^{*} & \sigma V_{\mathbf{h}}{}^{*} \\ \sigma V_{\mathrm{g}} & 2\pi\zeta_g & \sigma V_{\mathbf{h-g}}{}^{*} \\ \sigma V_{\mathbf{h}} & \sigma V_{\mathbf{h-g}} & 2\pi\zeta_h \end{pmatrix}, \tag{S2}$$

is the eigenmatrix for the equation:

$$\boldsymbol{M}\,C = \lambda_i\,C\,. \tag{S3}$$

The incident beam direction with respect to the crystal is described by the excitation errors of reflections g and h, denoted as ζ_g and ζ_h respectively. The structure factor, $V_{-\mathbf{g}}$, is replaced by $V_{\mathbf{g}}{}^{*}$ in the Hermitian matrix and σ is the interaction constant.

The 3x3 Hermitian matrix $\boldsymbol{M}$ has three real eigenvalues, $\lambda_1,\ \lambda_2,\ \lambda_3$, and each corresponds to a Bloch wave. The eigenvalues are calculated from the characteristic equation $|\boldsymbol{M} - \lambda_i \boldsymbol{I}| = 0$, which yields

$$\lambda^3 + b\lambda^2 + c\lambda + d = 0\ , \tag{S4a}$$

where $$b = -2\pi(\zeta_g + \zeta_h)\ ,\tag{S4b}$$

$$c = (2\pi\zeta_g)(2\pi\zeta_h) - (\sigma|V_{\mathbf{g}}|)^2 - (\sigma|V_{\mathbf{h}}|)^2 - (\sigma|V_{\mathbf{h-g}}|)^2\tag{S4c}$$

and $$d = (2\pi\zeta_g)(\sigma|V_{\mathbf{h}}|)^2 + (2\pi\zeta_h)(\sigma|V_{\mathbf{g}}|)^2 - 2(\sigma|V_{\mathbf{g}}|)(\sigma|V_{\mathbf{h}}|)(\sigma|V_{\mathbf{h-g}}|)cos\phi\ .\tag{S4d}$$

The real roots to this cubic equation can be expressed in trigonometric forms:

$$\lambda_1 = 2\sqrt{-Q}\,cos\left(\frac{\theta}{3}\right) - \frac{b}{3}\ ,\tag{S5a}$$

$$\lambda_2 = 2\sqrt{-Q}\,cos\left(\frac{\theta + 2\pi}{3}\right) - \frac{b}{3}\tag{S5b}$$

and $$\lambda_3 = 2\sqrt{-Q}\,cos\left(\frac{\theta + 4\pi}{3}\right) - \frac{b}{3}\ ,\tag{S5c}$$

where $$Q = \frac{3c - b^2}{9}\ ,\tag{S5d}$$

$$R = \frac{9bc - 27d - 2b^3}{54}\tag{S5e}$$

and $$\theta = cos^{-1}\left(\frac{R}{\sqrt{-Q^3}}\right)\ .\tag{S5f}$$

Fig. S1. shows a plot of the three dispersion surfaces along the Bragg condition for reflection g, which lies on a line (the so-called Bragg line) where $\zeta_g = 0$, and ζ_h varies continuously. Note: $\lambda_1, \lambda_2, \lambda_3$ have been assigned in a sequence such that $\lambda_1 \geq \lambda_2 \geq \lambda_3$.

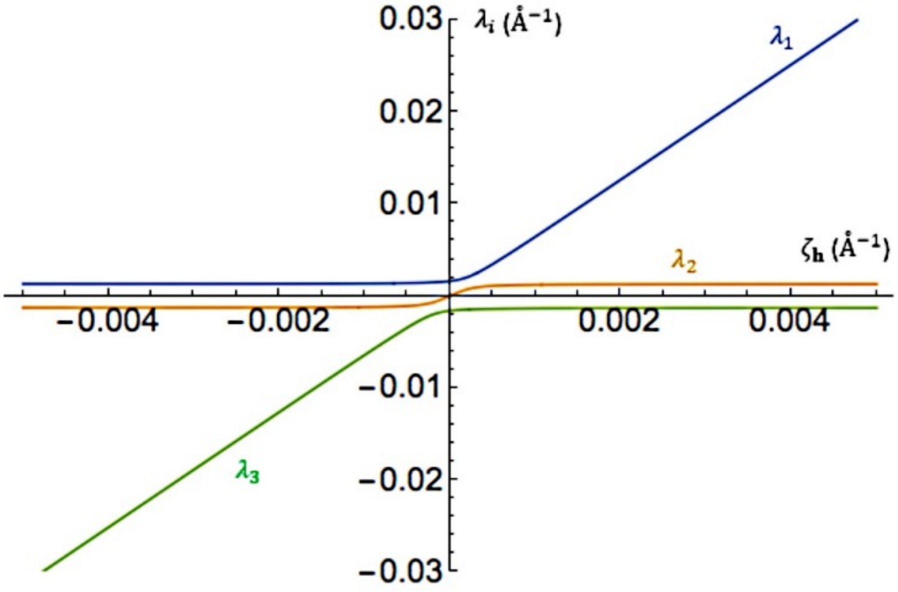

Figure S1 A plot of (the cut view of) dispersion surfaces in three-beam electron diffraction. The three eigenvalues, λ_1, λ_2, λ_3, are described by equations (S5a-c). Wolfram Mathematica 10 (Wolfram Research Inc., 2014) is used for generating this plot.

Following Hurley et al.(1978), we apply the projection operator

$$exp(i\,\boldsymbol{M}\,z) = \sum_{i=1}^{3} \boldsymbol{P_i}\,exp(i\,\lambda_i\,z) \quad \text{(S6a)}$$

and
$$\boldsymbol{P_i} = \frac{\boldsymbol{M} - \lambda_{i\oplus1}\boldsymbol{I}}{\lambda_i - \lambda_{i\oplus1}}\,\frac{\boldsymbol{M} - \lambda_{i\oplus2}\boldsymbol{I}}{\lambda_i - \lambda_{i\oplus2}} \quad (i = 1,2,3), \quad \text{(S6b)}$$

where an operator ⊕ is introduced to indicate cyclic addition: 1⊕1=2, 1⊕2=3, 2⊕1=3, 2⊕2=1, 3⊕1=1, 3⊕2=2. Before moving on, we introduce some shorthand notations:

$S_g = 2\pi\zeta_g$, $U_1 = \sigma|V_{\mathbf{g}}|$, $U_2 = \sigma|V_{\mathbf{h}}|$, $U_3 = \sigma|V_{\mathbf{h-g}}|$,

and $\mu_i = \lambda_{i\oplus1} - \lambda_{i\oplus2}$ (i.e. $\mu_1 = \lambda_2 - \lambda_3$, $\mu_2 = \lambda_3 - \lambda_1$, $and\ \mu_3 = \lambda_1 - \lambda_2$).

By combining equations (S1), (S5a, b) and (S6a, b), we can obtain the wave functions for reflections g and 0:

$$\psi_g = -U_1\,exp(i\varphi_{\mathbf{g}}) \sum_{i=1}^{3} exp(i\,\lambda_i z)\,\frac{\frac{U_2\,U_3}{U_1}exp(-i\phi) + S_{\boldsymbol{g}} - \lambda_{i\oplus1} - \lambda_{i\oplus2}}{\mu_{i\oplus1}\,\mu_{i\oplus2}} \quad \text{(S7)}$$

and
$$\psi_0 = -\sum_{i=1}^{3} exp(i\,\lambda_i z)\,\frac{\lambda_{i\oplus1}\lambda_{i\oplus2} + {U_1}^2 + {U_2}^2}{\mu_{i\oplus1}\,\mu_{i\oplus2}}\ . \quad \text{(S8)}$$

Thus, we can derive the intensity expressions for reflections g and h:

$$I_g = \psi_g\,\psi_g{}^* = 2{U_1}^2 \sum_{i=1}^{3} \{(cos\mu_i z - 1)\left(\frac{|G_0|^2}{\mu_1\mu_2\mu_3\mu_i} + G_{i\,\oplus\,1}G_{i\oplus2}\right)$$
$$+(cos(\mu_i z + \phi) - cos\phi)\,\frac{|G_0|G_{i\,\oplus\,1}}{\mu_i\;\mu_{i\,\oplus\,1}} +$$
$$(cos(\mu_i z - \phi) - cos\phi)\,\frac{|G_0|G_{i\oplus2}}{\mu_i\;\mu_{i\oplus2}}\} \quad \text{(S9a)}$$

and
$$I_h = \psi_h\,\psi_h{}^* = 2{U_2}^2 \sum_{i}^{3} \{(cos\mu_i z - 1)\left(\frac{|G_0{}'|^2}{\mu_1\mu_2\mu_3\mu_i} + G_{i+1}{}'G_{i+2}{}'\right)$$
$$+(cos(\mu_i z - \phi) - cos\phi)\,\frac{|G_0{}'|G_{i\,\oplus\,1}{}'}{\mu_i\;\mu_{i\,\oplus\,1}} +$$
$$(cos(\mu_i z + \phi) - cos\phi)\,\frac{|G_0{}'|G_{i\oplus2}{}'}{\mu_i\;\mu_{i\oplus2}}\}\,, \quad \text{(S9b)}$$

where
$$|G_0| = \frac{U_2\,U_3}{U_1}, |G_0{}'| = \frac{U_1\,U_3}{U_2}\ ;$$

and
$$G_i = \frac{S_g - \lambda_{i\oplus1} - \lambda_{i\oplus2}}{\mu_{i\oplus1}\,\mu_{i\oplus2}}\,, \qquad G_i{}' = \frac{S_h - \lambda_{i\oplus1} - \lambda_{i\oplus2}}{\mu_{i\oplus1}\,\mu_{i\oplus2}}\ .$$

The intensity expression for the central beam is:

$$I_0 = \psi_0\,\psi_0^{\,*} = 1 + 2\sum_{i=1}^{3} C_{i\oplus 1}C_{i\oplus 2}\,(cos\mu_i z - 1)\quad , \tag{S10}$$

where
$$C_i = \frac{\lambda_{i\oplus 1}\lambda_{i\oplus 2} + {U_1}^2 + {U_2}^2}{\mu_{i\oplus 1}\,\mu_{i\oplus 2}}.$$

From equations (S9a, b) and (S10), it can be seen that $I_{\boldsymbol{g}}$ and $I_{\boldsymbol{h}}$ depend on the three-phase invariant, ϕ, and $I_{\mathbf{0}}$ depends only on its magnitude, $|\phi|$. Here, the three-phase invariant is defined as the summation of three structure factor phases, $\phi \equiv \varphi_{\mathbf{g}} + \varphi_{\mathbf{h-g}} + \varphi_{\mathbf{-h}}$, and the reciprocal lattice vectors, **g**, **h-g**, **-h** form a closed loop in the anticlockwise direction, which sets up the sign convention[1] for three-phase invariants.

Although the expressions for I_g and I_h in equations (S9a, b) and (S10) convey all the structural parameters, an inversion for the three-phase invariant, ϕ, is still not straightforward. To invert the three-phase invariant, ϕ, or at least the signs of $sin\phi$ and $cos\phi$, it is necessary to reduce equation (S9) further. To invert the sign of $sin\phi$, S1.2 will reduce equation (S9a). To invert the sign of $cos\phi$ in a practical way, S1.3 will introduce some approximations when reducing equation (S9a).

S1.2. Derivation of equation (3)

Commencing from equation (S9a), one can derive a short expression for the intensity difference between a Friedel pair, g and $\overline{g}$. To be more specific, it is the intensity of g at a point (ζ_g, ζ_h) near the three-beam condition for 0 / g / h subtracted by the intensity of $\overline{g}$ at $(\zeta_{\overline{g}}, \zeta_{\overline{h}})$ = (ζ_g, ζ_h) near the three-beam condition for 0 / $\overline{g}$ / $\overline{h}$:

$$I_g(\zeta_g, \zeta_h, z) - I_{\overline{g}}(\zeta_{\overline{g}}, \zeta_{\overline{h}}, z) = -4\,sin\phi\; U_1U_2U_3\sum_{i=1}^{3}\frac{sin(\mu_i z)}{\mu_1\mu_2\mu_3}\ , \tag{S11a}$$

where ζ_g and ζ_h are the excitation errors for reflections g and h, and $\zeta_{\overline{g}}$ and $\zeta_{\overline{h}}$ are the excitation errors for reflections $\overline{g}$ and $\overline{h}$.

This is identical to equation (23) in the paper by Hurley *et al.* (1999).

Since $\mu_1 + \mu_2 + \mu_3 = 0$, equation (S11a) can be factorized:

$$I_g(\zeta_g, \zeta_h, z) - I_{\overline{g}}(\zeta_{\overline{g}}, \zeta_{\overline{h}}, z) = 16sin\phi\; U_1U_2U_3\prod_{i=1}^{3}\frac{sin(\frac{\mu_i z}{2})}{\mu_i}\ . \tag{S11b}$$

By replacing the shorthand notations with the original symbols, we obtain the form in equation (3).

S1.3. Derivation of equation (4)

If the three-phase invariant is defined as $\phi \equiv \varphi_{\mathbf{h}} + \varphi_{\mathbf{g-h}} + \varphi_{\mathbf{-g}}$, then the sign of ϕ will be flipped.

Mathematica (Wolfram Research Inc., 2014) has been used to generate interactive plots of the analytical functions of eigenvalues, intensities, some polynomials and so on, which allows for visualisation of these functions for all possible values of the input parameters. The Mathematica code can be obtained from the following website: https://github.com/DrYGuo/3-beam-project. There are five parameters in the input to these functions: $|V_{\mathbf{g}}|$, $|V_{\mathbf{h}}|$, $|V_{\mathbf{h-g}}|$, ϕ and z. A wide range of possible values has been set for each parameter: $|V_{\mathbf{g}}|, |V_{\mathbf{h}}|, |V_{\mathbf{h-g}}| \in [0,7]$ (in V), $\phi \in [-\pi, \pi]$, and $z \in [0, 2000]$(in Å).

In some situations, it is difficult, if not impossible, to derive a mathematical relation, especially an approximate equality by using pure mathematics alone. In the current practical problem in which finding the features that relate to the sign of cosine three-phase invariants is the main concern, we allow the use of some empirical rules such as approximate equalities that are concluded from observations of the interactive plots: if an approximate equality is always found in the interactive plots where the five parameters have been finely tuned to give almost all the possible combinations of their values within their ranges, then it can be accepted as an empirical rule that such an approximate equality holds for three-beam dynamic diffraction in all cases. For the purpose of deriving empirical rules, the interactive plots will be used in the current and next sections.

S1.3.1. Derivation of the criteria for determining the sign of $cos\phi$

In equation (S9a) or (S10), either I_g or I_0 is a summation of three polynomials. An interactive plot is generated for the three polynomials in equation (S9a) along the Bragg line of reflection g (an example is shown in Fig.S2). The interactive plots show that a certain distance[2], $|\Delta\zeta_{\boldsymbol{h}}|^c$, away from the exact three-beam condition, $\zeta_{\boldsymbol{h}} = 0$, the intensity is mostly contributed by only one of the three polynomials. The distinct contributions of the polynomials can be explained by the terms $1/(\mu_1\mu_2\mu_3\mu_i)$ in their expressions, which include the dispersion surface gaps μ_i in the denominator: when $\zeta_h < -|\Delta\zeta_h|^c$,$|\mu_3| \ll |\mu_1| < |\mu_2|$; when $\zeta_h > |\Delta\zeta_h|^c, |\mu_1| \ll |\mu_3| < |\mu_2|$ (which can be seen from Fig. S3). Therefore, we can approximate the exact solution, which is a summation of three polynomials, by only one of the three polynomials for the regions away from the exact three-beam condition. By factorisation of the first and the third polynomial (i =1 and 3), we can derive a piecewise function that is asymptotic to the exact solutions given by equations (S9a) and (S10) on both sides of the three-beam condition:

$$I_g \approx \begin{cases} (cos\mu_3 z - 1)\, T_3, & for\ \zeta_h < -|\Delta\zeta_h|^c \\ (cos\mu_1 z - 1)\, T_1, & for\ \zeta_h > \ |\Delta\zeta_h|^c \end{cases} \qquad \text{(S12a)}$$

[2] The value of $|\Delta\zeta_h|^c$ is very small for reflections having small structure factor magnitudes but increases with increasing structure factor magnitudes.

and $$I_0 \approx \begin{cases} 1 + 2C_1C_2(cos\mu_3 z - 1), & for\ \ \zeta_h < -|\Delta\zeta_h|^c \\ 1 + 2C_3C_2(cos\mu_1 z - 1), & for\ \ \zeta_h > |\Delta\zeta_h|^c \end{cases}, \tag{S12b}$$

where the terms[3], T1 and T3, are independent of the thickness, z, and

$$T_3 = \frac{2\ U_1{}^2}{\mu_1\mu_2\mu_3{}^2}\{G_0{}^2 - G_0(\lambda_1 + \lambda_2 + 2\lambda_3)cos\phi + (\lambda_1 + \lambda_3)(\lambda_2 + \lambda_3)\} \tag{S13a}$$

and $$T_1 = \frac{2\ U_1{}^2}{\mu_1{}^2\mu_2\mu_3}\{G_0{}^2 - G_0(\lambda_2 + \lambda_3 + 2\lambda_1)cos\phi + (\lambda_2 + \lambda_1)(\lambda_1 + \lambda_3)\}. \tag{S13b}$$

The interactive plots of $T_1\ (\zeta_h)$, $T_3\ (\zeta_h)$, $C_1(\zeta_h)C_2(\zeta_h)$ and $C_2(\zeta_h)C_3(\zeta_h)$ show that

$$T_3\ (-|\zeta_h|) \approx T_1\ (|\zeta_h|), \quad for\ \ |\zeta_h| > |\Delta\zeta_h|^c; \tag{S14a}$$

and $$C_1(-|\zeta_h|)C_2(-|\zeta_h|) \approx C_2(|\zeta_h|)C_3(|\zeta_h|), \quad for\ \ |\zeta_h| > |\Delta\zeta_h|^c\ . \tag{S14b}$$

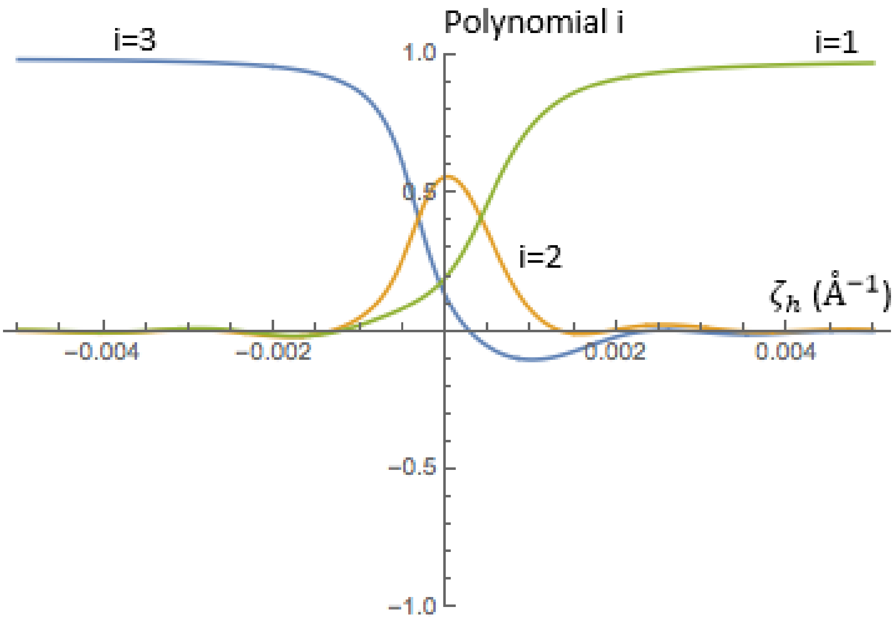

Figure S2 Plots of the three polynomials in equations (S5a-c) along the Bragg line of reflection g. The summation of the three gives the intensity profile of I_g along its Bragg line. On the left hand side (say $\zeta_h < -0.002$ Å^{-1}), the summation of the three polynomials is governed by the polynomial where i =3 while on the right hand side (say $\zeta_h > 0.002$ Å^{-1}), the summation of the three polynomials is governed by the polynomial where i =1. Wolfram Mathematica 10 (Wolfram Research Inc., 2014) was used for this plot.

[3] A term involving sinϕ has been ignored because its contribution in the region $|\zeta_h| > |\Delta\zeta_h|^c$ is very small.

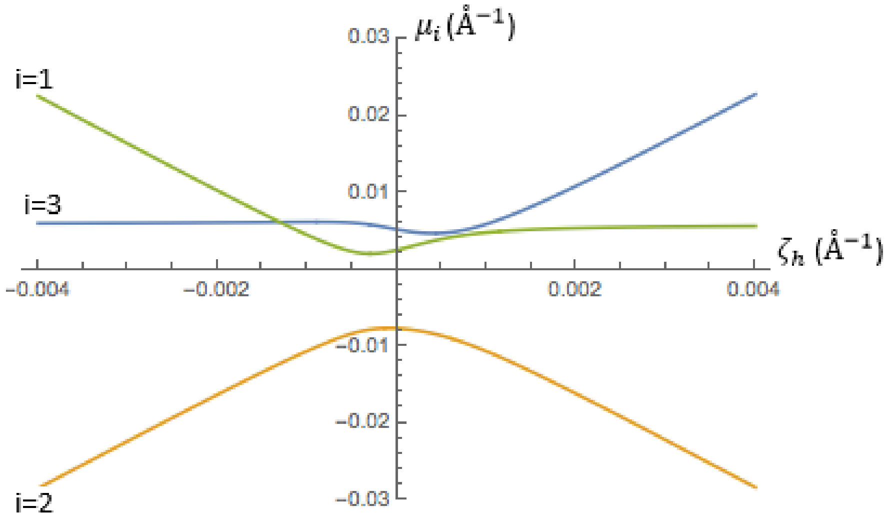

Figure S3 The gaps of the three dispersion surfaces, μ_i, along the Bragg line of g. On the left hand side (say $\zeta_h < -0.002$ Å^{-1}), $|\mu_3| \ll |\mu_1| < |\mu_2|$; while on the right hand side (say $\zeta_h > 0.002$ Å^{-1}), $|\mu_1| \ll |\mu_3| < |\mu_2|$. Wolfram Mathematica 10 (Wolfram Research Inc., 2014) was used for this plot.

Combining equations (S12a) and (S14a), we have

$$\frac{I_g(-|\zeta_h|)}{I_g(|\zeta_h|)} \approx \frac{sin^2(\mu_3 z/2)}{sin^2(\mu_1 z/2)}\ , \qquad \text{for } |\zeta_h| > |\Delta\zeta_h|^c \left(\text{except for } z \approx \frac{2\pi}{\mu_1}\right). \qquad \text{(S15)}$$

In other words, the asymmetry between $I_g(-|\zeta_h|)$ and $I_g(|\zeta_h|)$ as well as between $I_0(-|\zeta_h|)$ and $I_0(|\zeta_h|)$ is dominated by the difference between the thickness-dependent factors $sin^2(\mu_3 z/2)$ and $sin^2(\mu_1 z/2)$. That completes the derivation of equation (4).

S2. Derivation of Criterion 2

Valid range of thickness for direct observation of the sign of sinϕ

To decide whether the thickness is smaller than the “three-beam extinction distance”, ξ_{3-beam}, inspection of the Bragg lines is needed. Two conditions listed in criterion 2 must be satisfied.
From equation (3), we can see that the intensity difference between a Friedel or Bijvoet pair will be small if the thickness gets close to the three-beam extinction distance, ξ_{3-beam}. To have a contrast that is observable even by eye, the thickness has to differ from the three-beam extinction distance by a certain value. As an empirical rule, when $z < 0.85\ \xi_{3-beam}$ or $z > 1.15\ \xi_{3-beam}$ (except for very thick specimens, which can be easily recognised from observing the corresponding CBED patterns), then the contrast is usually observable by eye. Therefore, as long as one can distinguish the CBED patterns for $z < 0.85\ \xi_{3-beam}$ from those for $z > 1.15\ \xi_{3-beam}$, one will be able to decide whether

the thickness is smaller than the extinction distance, ξ_{3-beam}. Here, we are able to find two conditions, I and II. The satisfaction of both conditions is sufficient but not necessary for having $z < \xi_{3-beam}$. The two conditions are derived from two opposite situations, as will be discussed below.

S2.1. When max $\{|\boldsymbol{V}_{\mathbf{g}}|, |\boldsymbol{V}_{\mathbf{h}}|\} \gtrsim |\boldsymbol{V}_{\mathbf{h-g}}|$

The curves, $\mu_1(\zeta_h)$, $\mu_3(\zeta_h)$ along the Bragg line of g (e.g. in Fig. S3) always have local minima near the exact three-beam conditions, $\zeta_h = 0$. Thus, we have

$$|\mu_1(\zeta_h = 0)| < \mu_1(\zeta_h > |\Delta\zeta_h|^c)\,, \quad \text{(S16a)}$$

$$|\mu_3(\zeta_h = 0)| < \mu_3(\zeta_h < -|\Delta\zeta_h|^c) \quad \text{(S16b)}$$

and

$$|\mu_2(\zeta_h = 0)| < \mu_3(\zeta_h < -|\Delta\zeta_h|^c) + \mu_1(\zeta_h > |\Delta\zeta_h|^c) \quad \text{(S16c)}$$

Without losing generality, we consider the case for max$\{|V_{\mathbf{g}}|, |V_{\mathbf{h}}|\} = |V_{\mathbf{g}}|$:

for $cos\phi > 0$,

$$\xi_{3-beam} = \frac{2\pi}{|\mu_2(\zeta_h = 0)|} \gtrsim \frac{1}{2}\frac{2\pi}{|\mu_3(\zeta_h < -|\Delta\zeta_h|^c)|},$$

$$=> \xi_{3-beam} \gtrsim \frac{1}{2}\xi_{pseudo-2beam}(\zeta_h < -|\Delta\zeta_h|^c)\,; \quad \text{(S17a)}$$

for $cos\phi < 0$,

$$\xi_{3-beam} = \frac{2\pi}{|\mu_2(\zeta_h = 0)|} \gtrsim \frac{1}{2}\frac{2\pi}{|\mu_1(\zeta_h > |\Delta\zeta_h|^c)|},$$

$$=> \xi_{3-beam} \gtrsim \frac{1}{2}\xi_{pseudo-2beam}(\zeta_h > |\Delta\zeta_h|^c)\,, \quad \text{(S17b)}$$

where $\xi_{pseudo-2beam}(\zeta_h < -|\Delta\zeta_h|^c)$ denotes the two-beam extinction distance for the pseudo-2beam condition regions (i.e. along the Bragg condition of $\zeta_g = 0$ but away from the three-beam condition) on the negative side of ζ_h.

Here, a shorthand notation is introduced:

$$\xi_{pseudo-2beam} = min\left\{\xi_{pseudo-2beam}\left(\zeta_h < -|\Delta\zeta_h|^c\right), \xi_{pseudo-2beam}\left(\zeta_h > |\Delta\zeta_h|^c\right)\right\}.$$

Then the inequalities (S17a, b) can be re-written as:

$$\xi_{3-beam} \gtrsim \frac{1}{2}\xi_{pseudo-2beam}. \quad \text{(S17c)}$$

According to the two-beam diffraction equations (for example, Blackman, 1939, Spence & Zuo, 1992), when $z = \frac{1}{2}\,\xi$, the central bright peak is 1.618 times as broad as the neighbouring bright peak. Therefore, when max$\{|V_{\mathbf{g}}|, |V_{\mathbf{h}}|\} \gtrsim |V_{\mathbf{h-g}}|$, $z < \frac{1}{2}\xi_{pseudo-2beam}$ is a sufficient condition for $z \lesssim \xi_{3-beam}$ and condition I can be stated as:

Condition I: The excess Bragg line in the "pseudo two-beam regions" of the stronger reflection has a central bright fringe that is at least 1.6 times wider than the second bright fringe.

That completes the proof for condition I.

Next, we consider the opposite situation.

S2.1.1. When max$\{|\boldsymbol{V}_{\mathbf{g}}|, |\boldsymbol{V}_{\mathbf{h}}|\} \ll |\boldsymbol{V}_{\mathbf{h-g}}|$

In such a situation, the interactive plots of $\mu_i(\zeta_h)$ along the Bragg line of reflection g, $\zeta_g = 0$, show that

$$|\mu_2(\zeta_h = 0)| > \mu_3(\zeta_h < -|\Delta\zeta_h|^c) + \mu_1(\zeta_h > |\Delta\zeta_h|^c)\,. \tag{S18}$$

Now, condition I is no longer sufficient for $z < \xi_{3-beam}$, so we need to derive another sufficient condition for this situation.

Fig. S4a shows the plot of $C_{i\oplus1}C_{i\oplus2}$, the terms in equation (S10), along the locus $\zeta_g = \zeta_h$, where $C_1C_3 \approx 0$ (an empirical rule), C_2C_3 and C_1C_2 show two peaks at the turning points of μ_3 and μ_1 on each side of $\zeta_h = 0$. These turning points are close to the exact three-beam condition, $\zeta_h = 0$ (shown in Fig. S4b).

We first consider a case when $|\phi| = \frac{\pi}{2}$. Since $C_1C_3 \approx 0$, equation (S10) becomes:

$$I_0 = 1 + 2C_2C_3(cos\mu_1 z - 1) + 2C_1C_2(cos\mu_3 z - 1)\,. \tag{S19}$$

Here we consider any point on the locus, $\zeta_g = \zeta_h$, that is <u>close to the three-beam condition, $\zeta_h = 0$, from the negative side (shown by the dashed line in Figs. S4 & 5)</u>. All the arguments or functions below refer to the same point, and the coordinate, (ζ_h), will be omitted for short-hand notations. The derivative of I_0 with respect to ζ_h is:

$$\frac{dI_0}{d\zeta_h} = 2\frac{dC_2C_3}{d\zeta_h}(cos\mu_1 z - 1) + 2\frac{dC_1C_2}{d\zeta_h}(cos\mu_3 z - 1) - 2C_2C_3 sin\mu_1 z\frac{d\mu_1 z}{d\zeta_h} - 2C_1C_2 sin\mu_3 z\frac{d\mu_3 z}{d\zeta_h}\,. \tag{S20a}$$

Since $$\left|\frac{dC_2C_3}{d\zeta_h}\right| \gg \left|C_2C_3\frac{d\mu_1}{d\zeta_h}\right|,$$

We have $$\frac{dI_0}{d\zeta_h} \approx 2\frac{dC_2C_3}{d\zeta_h}(cos\mu_1 z - 1) + 2\frac{dC_1C_2}{d\zeta_h}(cos\mu_3 z - 1)\,. \tag{S20b}$$

For any thickness, we have:

$$2\frac{dC_2C_3}{d\zeta_h}(cos\mu_1 z - 1) > 0 \ , \quad \text{(S21a)}$$

$$2\frac{dC_1C_2}{d\zeta_h}(cos\mu_3 z - 1) < 0 \, , \quad \text{(S21b)}$$

$$\text{and } \left|\frac{dC_1C_2}{d\zeta_h}\right| < \left|\frac{dC_2C_3}{d\zeta_h}\right| . \quad \text{(S22)}$$

For very thin specimens, where the perturbation theory of kinematic diffraction can be applied, we have

$$\frac{dI_0}{d\zeta_h} < 0 \, , \quad \text{(S23a)}$$

which is equivalent to

$$\frac{|\frac{dC_1C_2}{d\zeta_h}|}{|\frac{dC_2C_3}{d\zeta_h}|}\frac{|sin^2\left(\frac{\mu_3 z}{2}\right)|}{|sin^2\left(\frac{\mu_1 z}{2}\right)|} > 1 \quad \text{(S23b)}$$

In other words, $\frac{|sin^2\left(\frac{\mu_3 z}{2}\right)|}{\left|sin^2\left(\frac{\mu_1 z}{2}\right)\right|}$ (>1) beats the multiplier $\frac{\left|\frac{dC_1C_2}{d\zeta_h}\right|}{\left|\frac{dC_2C_3}{d\zeta_h}\right|}$ (<1) and results in a product of larger than 1 for very thin specimens. When the thickness increases, $\frac{\left|\frac{dC_1C_2}{d\zeta_h}\right|}{\left|\frac{dC_2C_3}{d\zeta_h}\right|}$ is constant while $\frac{|sin^2\left(\frac{\mu_3 z}{2}\right)|}{\left|sin^2\left(\frac{\mu_1 z}{2}\right)\right|}$ will decrease from a value of larger than 1 to a value of less than 1 and the product, and thus $\frac{|\frac{dC_1C_2}{d\zeta_h}|}{|\frac{dC_2C_3}{d\zeta_h}|}\frac{|sin^2\left(\frac{\mu_3 z}{2}\right)|}{|sin^2\left(\frac{\mu_1 z}{2}\right)|}$, will become less than 1, and $\frac{dI_0}{d\zeta_h}$ will become positive at a certain stage. The sign of $\frac{dI_0}{d\zeta_h}$ will be flipped when the thickness increases to about:

$$\frac{\mu_1 z}{2} = \frac{\pi}{2} . \quad \text{(S24)}$$

From the interactive plots (an example of the plots is given in Fig. S4b), we have

$$\mu_1 \approx \frac{|\mu_2(\zeta_h = 0)|}{2} . \quad \text{(S25)}$$

Therefore,

$$z \geq \xi_{3-beam} \equiv \frac{2\pi}{\mu_2(\zeta_h = 0)} \approx \frac{\pi}{\mu_1}$$

$$=> \frac{dI_0}{d\zeta_h} > 0 \, . \quad \text{(S26a)}$$

The converse-negative proposition is also true:

$$\frac{dI_0}{d\zeta_h} \leq 0 \ => \ z < \xi_{3-beam} \,. \tag{S26b}$$

Now, we consider any point on the locus, $\zeta_g = \zeta_h$, that is close to the three-beam condition, $\zeta_h = 0$, from the positive side. Similarly, we have:

$$\frac{dI_0}{d\zeta_h} \geq 0 \ => \ z < \xi_{3-beam} \,. \tag{S26c}$$

In summary, for any point that is along the locus $\zeta_g = \zeta_h$ and near the three-beam condition, we have:

$$\frac{d^2 I_0}{d{\zeta_h}^2} \geq 0 => \ z < \xi_{3-beam} \,. \tag{S26d}$$

Similar arguments can be repeated for $|\phi| \neq \frac{\pi}{2}$, where the sign of $\frac{dI_0}{d\zeta_h}$ near the point that corresponds to the intersection of C_2C_3 and C_1C_2 (rather than $\zeta_h = 0$) is considered.

Therefore, we can state condition II:

Condition II: Near the exact three-beam condition in the central beam, the intensity along the locus $\zeta_g = \zeta_h$ has a local minimum, i.e. $\frac{d^2 I_0}{d{\zeta_h}^2} \geq 0$, then $z < \xi_{3-beam}$.

When both conditions I and II are satisfied, it is sufficient to conclude that the thickness is smaller than the three-beam extinction distance or the maximum allowed thickness for direct observation of the sign. Then, the sign of the three-phase invariants can be determined by comparing the intensity difference directly without measuring the thickness. Otherwise, without the knowledge of the thickness and the structure factor magnitudes, the diffraction pattern should be rejected as unsuitable for the direct determination of the sign by inspection.

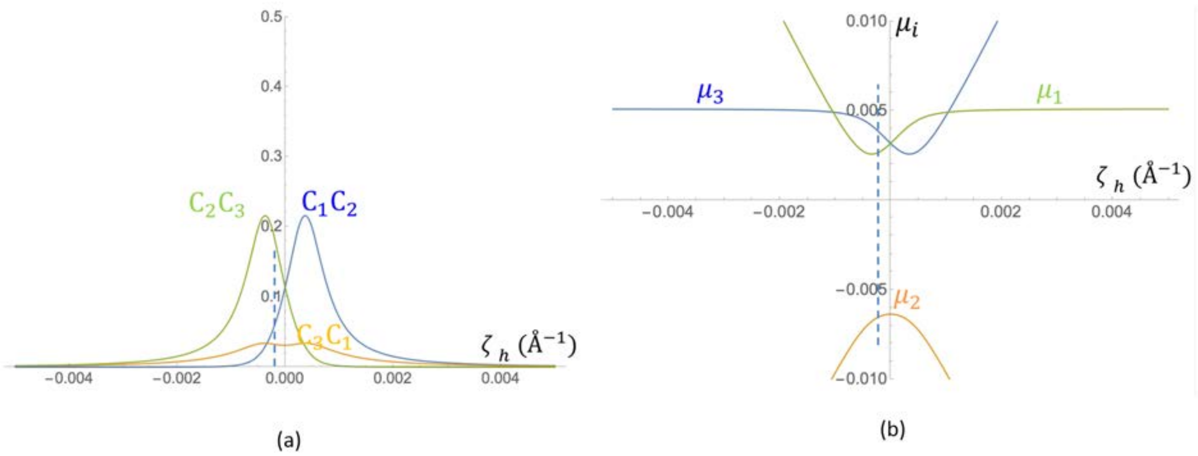

Figure S4 Plots of (a) the polynomials $C_{i\oplus1}C_{i\oplus2}$ from equation (3.10) and (b) the gaps of the dispersion surfaces, μ_i, versus the excitation error ζ_h along the locus, $\zeta_g = \zeta_h$ (the horizontal axis). The dashed line shows a point that can be anywhere close to the three-beam condition, $\zeta_h = 0$, from the negative side, $\zeta_h < 0$. Wolfram Mathematica 10 (Wolfram Research Inc., 2014) was used for generating this plot.

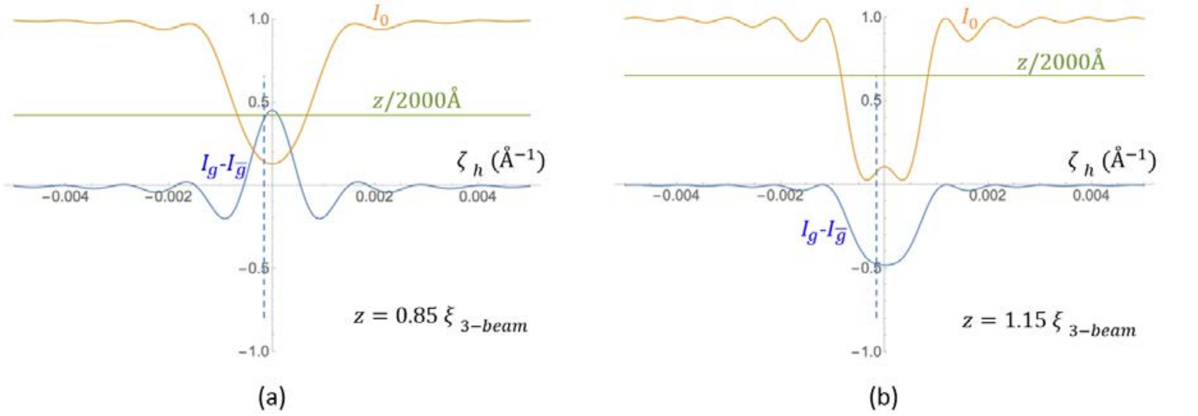

Figure S5 Plots of the intensity of the central beam (in orange) and the intensity difference between a Friedel pair, g and $\overline{g}$, (in blue) along the locus $\zeta_g = \zeta_h$ (the horizontal axes). When the thickness increases from (a) $z = 0.85\ \xi_{3-beam}$ to (b) $z = 1.15\ \xi_{3-beam}$, the sign of the intensity difference flips to the opposite direction and the convexity of intensity profile of the central beam, I_0, is also changed. The dashed line shows a point that can be anywhere close to the three-beam condition, $\zeta_h = 0$, from the negative side, $\zeta_h < 0$. Wolfram Mathematica 10 (Wolfram Research Inc., 2014) was used for generating these plots.

References

Blackman, M. (1939). *Proc. R. Soc. Lon. Ser.* A**173**, 0068-0082.

Hurley, A. C., Johnson, A. W. S., Moodie, A. F., Rez, P. & Sellar, J. R. (1978). *Inst. Phys. Conf. Ser.* No. 41, pp. 34-40.

Hurley, A. C., Moodie, A. F., Johnson, A. W. & Abbott, P. C. (1999). *Acta Cryst.* A**55**, 216-219.

Wolfram Research, Inc. (2014). Mathematica. Version 10.0. Wolfram Research, Inc., Champaign, Illinois.

Spence, J. C. H. & Zuo, J. M. (1992). *Electron microdiffraction*. New York: Plenum Press.
